# Membrane and Soluble CD137 in Systemic Lupus Erythematosus: Role as Biomarkers for Disease Activity

**DOI:** 10.1155/2023/2344239

**Published:** 2023-04-18

**Authors:** Fulvia Ceccarelli, Francesco Natalucci, Alessandra Di Filippo, Giulio Olivieri, Chiara Napoletano, Aurelia Rughetti, Marianna Nuti, Ilaria Grazia Zizzari, Fabrizio Conti

**Affiliations:** ^1^Lupus Clinic, Rheumatology Unit, Department of Clinical, Internal, Anesthesiological and Cardiovascular Sciences, Sapienza University of Rome, Rome, Italy; ^2^Laboratory of Tumor Immunology and Cell Therapy, Department of Experimental Medicine, Policlinico Umberto I, Sapienza University of Rome, Rome, Italy

## Abstract

**Objective:**

The role of T cells in the pathogenesis of systemic lupus erythematosus (SLE) has recently gained attention. Costimulatory molecules are membrane proteins strictly associated with T-cell receptor (TCR), acting by activating or inhibiting T cells and antigen-presenting cells (APC) through direct and reverse signaling, thus becoming responsible for the development of effector T cells or regulatory T cells. The primary objective of the present case–control study was to evaluate the cell membrane expression of CD137 on T cells and the serum concentration of CD137 (sCD137) in a SLE cohort.

**Materials:**

We enrolled SLE patients and sex/age-matched healthy subjects (HS). Disease activity was assessed by SLEDAI-2K. By application of flow cytometry, we evaluated the expression of CD137 on CD4+ and CD8+ lymphocytes. ELISA test was performed to evaluate serum levels of sCD137.

**Results:**

Twenty-one SLE patients (M/F 1/20; median age 48 years (IQR 17); median disease duration 144 months (IQR 204)) were evaluated. SLE patients showed %CD3+CD137+ cells significantly higher compared to HS (median 5.32 (IQR 6.11) versus 3.3 (IQR 1.8), *p* = 0.001). In SLE patients, %CD4+CD137+ cells positively correlated with SLEDAI-2K (*p* = 0.0082, *r* = 0.58, CI (0.15–0.82); indeed, %CD4+CD137+ cells were significantly lower in SLE patients with a remission status compared to those not reaching this condition (median 1.07 (IQR 0.91) versus 1.58 (IQR 2.42), *p* = 0.013). Accordingly, sCD137 levels were significantly lower in remission status (31.30 pg/mL (IQR 102.2 versus median 122.8 pg/mL (IQR 536); *p* = 0.03) and correlated with %CD4+CD137+ cells (*p* = 0.012, *r* = 0.60, CI (0.15–0.84)).

**Conclusion:**

Our results suggest a possible involvement of CD137-CD137L axis in SLE pathogenesis, as demonstrated by higher expression of CD137 on CD4+ cells in SLE compared with HS. Furthermore, the positive correlation between SLEDAI-2K and membrane CD137 expression on CD4+ cells, as well as soluble CD137, indicates a possible use as biomarkers for disease activity.

## 1. Introduction

Systemic lupus erythematosus (SLE) is a chronic autoimmune disease with a wide spectrum of clinical manifestations, potentially involving the skin, joints, kidneys, and central nervous system (CNS) [1]. SLE is characterized by a deep immune system dysregulation, starting from autoantigen presentation to autoantibody production and tissue damage. Cellular communication, involving costimulatory molecules such as OX40, CD40, CD27, ICOS and PD-1, seems to play a key role in this pathogenic process [2]. Indeed, costimulation is a secondary signal involved in initiating, maintaining, and regulating immune reactions; its dysfunction results in abnormal immune responses. Several costimulatory pathways have been shown to be involved in SLE development. Thus, CD28^−/−^ MRL-lpr mice show milder disease [3], OX40:OX40L interaction may contribute to T follicular helper (Tfh) activation in SLE patients [4], while the CD40:CD40L axis seems to be able to promote B cells class switching in SLE murine model [5].

Among costimulatory molecules, CD137 (4-1BB) has been implicated in the dysregulation of costimulation mechanisms [6]. CD137 is a membrane-bound member of the tumor necrosis factor (TNF) receptor superfamily, prevalently expressed on activated CD4+ and CD8+ T cells [7]. By binding its ligand CD137L, expressed on antigen-presenting cells (APC), CD137 determines T cells activation and proliferation [8]. Furthermore, CD137 molecule is also released in the blood in soluble form (sCD137), generated by differential splicing [9, 10]. The function of sCD137 seems to be opposite to membrane-bound molecule. The interaction of sCD137 with CD137L blocks, in fact, the maturation of APCs and prevents the activation of T cells. In the scenario of autoimmunity, the role of CD137 expressed on T cells is controversial, without conclusive results about its negative or positive action on autoimmune disease development and phenotype [11].

To the best of our knowledge, the contribution of both membrane and soluble CD137 in SLE patients has not been investigated. Thus, this study aims to evaluate the role of CD137 in a SLE cohort to assess the possible association between membrane and serum CD137 and disease activity and phenotype.

## 2. Patients and Methods

We enrolled consecutive SLE patients (2019 American College of Rheumatology/European League Against Rheumatism criteria) [12] attending the Lupus Clinic of Sapienza University of Rome. As previously described, clinical, demographic, and laboratory of each enrolled patient were collected in a standardized computer-filled form, including date of diagnosis, previous and ongoing treatment, determination of autoantibodies (antinuclear antibodies, anti-dsDNA, extractable nuclear antigen, anti-SSA, anti-SSB, anti-Sm, anti-ribonucleoprotein, anticardiolipin (aCL), anti-*β*2glycoprotein I (ELISA), lupus anticoagulant (LA) (according to International Society of Thrombosis and Hemostasis), and C3/C4 serum levels (mg/dL, nephelometry) [13]. Disease activity has been evaluated by Systemic Lupus Erythematosus Activity Disease Index-2K (SLEDAI-2K) and chronic damage by SLICC Damage Index (SDI). To discriminate patients with different levels of disease activity, they were distinguished according to the presence of remission status defined as a stable disease in patients with no clinical or serological activity allowing therapy with hydroxychloroquine, immunosuppressant and/or biological therapy, and a daily prednisone equivalent dose <5 mg [14].

As control, we enrolled healthy donors (HDs) sex- and age-matched. This study was conducted according to the Declaration of Helsinki statements and was approved by the Ethics Committee of Policlinico Umberto I, Sapienza University of Rome (protocol number 26/3/2015). Informed consent was obtained from all the patients.

### 2.1. Isolation of Peripheral Blood Mononuclear Cells (PBMCs) and Serum Collection

Peripheral blood mononuclear cells (PBMCs) were isolated from patients and HDs using Ficoll–Hypaque density gradient separation. Patient's blood samples were first collected into EDTA anticoagulant tubes (BD Vacutainer) and then processed within 1 hr after blood sampling to isolate and collect PBMCs that were cryopreserved until use. Simultaneously, serum samples were collected and stored at −80°C by using Vacutainer Blood Collection Tubes.

### 2.2. Immunophenotyping Analysis

A multi-parametric cytofluorimetric analysis was conducted to evaluate the surface expression of CD137 on T cells subset. PBMCs were stained using the following conjugated anti-human monoclonal antibodies: anti-CD3 BV510 (clone HIT3a, catalog no.: 564713), anti-CD8 APC-H7 (clone SK1, catalog no.: 561423), and anti-CD137 APC (clone 4B4-1, catalog no.: 550890). Samples were acquired by FACSCanto II Flow Cytometer and analyzed by FACS Diva Software (version 8.0.2, BD Biosciences, San Diego, CA, USA) and FlowJo (version 10.8.8, Becton Dickinson) analysis software.

### 2.3. sCD137 Detection

Serum samples derived from SLE patients and HDs were used to measure the concentration of the soluble form of CD137 through the Human 4-1BB/TNFRS9 DuoSet Elisa Kit and the DuoSet Ancillary Reagent Kit (catalog #DY838 and #DY008, respectively, R&D System, Minneapolis, MN, USA) according to the manufacturer's instructions. The concentration of sCD137 was evaluated using Absorbance 96 Plate Reader (Byonoy) at 450 nm of absorbance.

### 2.4. Statistical Analysis

Version 9.0 of the GraphPad statistical package was used for statistical analysis. Normally distributed variables were summarized using the median ± IQR, and nonnormally distributed variables by the median and range. Frequencies were expressed by percentage. Wilcoxon's matched pairs test and paired *t*-test were performed accordingly. Univariate comparisons between nominal variables were calculated using the *χ*^2^ test or Fisher's exact test where appropriate. Spearman's test was used to assess the correlations. Two-tailed *p*-values were reported, and *p*-values <0.05 were considered as statistically significant. Grubbs' test has been performed to identify outliers values.

## 3. Results

### 3.1. Patient's Characteristics

We enrolled 20 SLE patients (19 F/1 M, median age 48 years (IQR 17), median disease duration 144 months (IQR 204)). Clinical and laboratory data and ongoing treatment are given in Table 1. At the time of enrollment, mucocutaneous involvement was the most frequent disease-related manifestation (six patients, 30%), followed by joint involvement (five patients, 25%) and lupus nephritis (four patients, 20%); moreover, seven patients (35%) had hypocomplementemia and five patients (25%) were positive for anti-dsDNA. Indeed, median SLEDAI-2K value was 2 (IQR 7.5) and 10 patients were in remission, according to the above-reported definition [13]. Furthermore, SDI median disease value was 0 (IQR 1).

Furthermore, Table 2 shows details about all the SLE patients enrolled in the present analysis. In particular, we included ongoing clinical and laboratory manifestations concurring to SLEDAI-2K values.

### 3.2. High Circulating Levels of CD4+CD137+ T Cells Are Associated with a Worse Clinical Outcome

**Figure 1:**
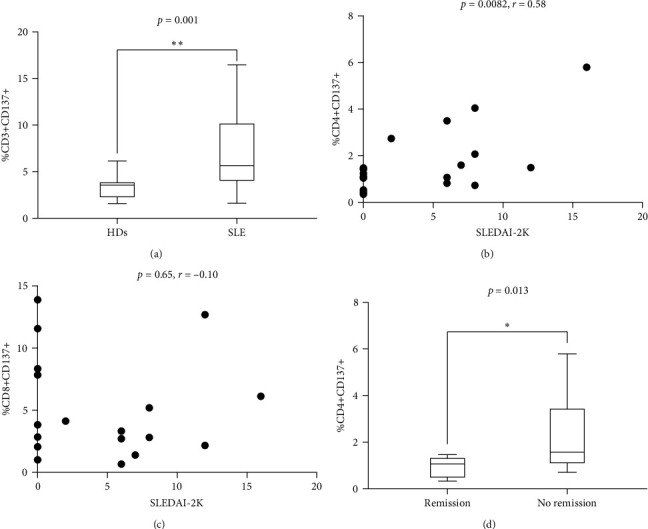
(a) Comparison of %CD3+CD137+ in HDs and SLE patients; (b) correlation between disease activity and %CD4+CD137+; (c) correlation between disease activity and %CD8+CD137+; (d) comparison of %CD4+CD137+ SLE patients in remission versus no remission patients.

Flow cytometry analysis evaluated the expression of CD137 on CD3+, CD4+, and CD8+ subset on SLE patients and HDs. As shown in Figure 1(a), the percentage of CD3+CD137+ T cells in SLE patients was significantly higher compared to HDs (median 5.32 (IQR 6.11) versus 3.3 (IQR 1.8), *p* = 0.001; Figure 1(a)). The frequency of CD137+ T cells was investigated in SLE patients; according to disease activity, as expressed by SLEDAI-2K, we identified a significant correlation between CD4+CD137+ T cells (*p* = 0.0082, *r* = 0.58, CI (0.15–0.82); Figure 1(b)). Differently, we have not observed any correlation between the percentage of CD8+CD137+ T cells and SLEDAI-2K values (*p* = 0.65, *r* = –0.10, CI (−0.54–0.37); Figure 1(c)), suggesting a peculiar role for CD4+ subset.

These results were also confirmed when distinguishing SLE patients according to the achievement of remission. Indeed, we observed a significantly lower percentage of CD4+CD137+ T cells in patients in remission compared with those who did not reach this condition (median 1.07 (IQR 0.91) versus 1.58 (IQR 2.42), *p* = 0.013; Figure 1(c)). No significant differences were observed by comparing the percentage of CD8+CD137+ T cells patients according to remission achievement (remission: median 5.8 (IQR 8.52) versus no remission: 2.8 (IQR 3.0), *p* = 0.31) (figure not shown).

### 3.3. Soluble CD137 Levels in Serum of SLE Patients Are Correlated with Disease Activity

**Figure 2:**
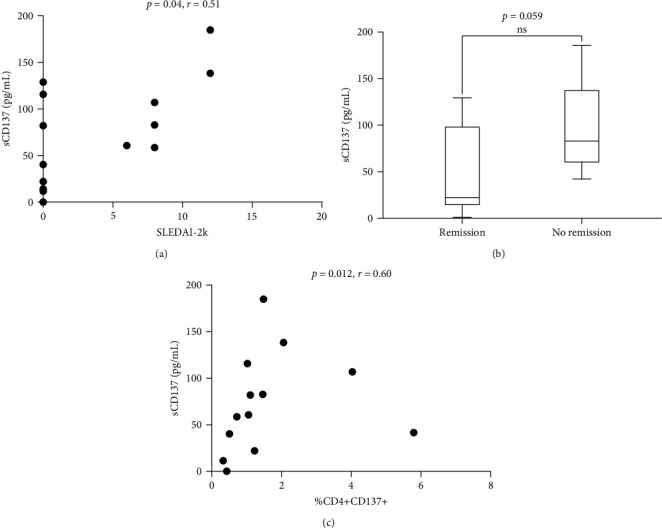
(a) Correlation between sCD137 serum levels and SLEDAI-2K; (b) comparison of sCD137 in SLE patients according to remission status; (c) correlation between sCD137 levels and %CD4+CD137+.

We found a positive correlation between sCD137 levels and disease activity assessed by SLEDAI-2K (*p* = 0.04, *r* = 0.51, CI (0.01–0.79)) (Figure 2(a)). Moreover, these levels were lower in patients in remission (22.15 pg/mL, IQR 102.2) in comparison with those who did not reach this condition (82.95 pg/mL, IQR 536, *p* = 0.059; Figure 2(b)). Finally, sCD137 serum levels were positively correlated with %CD4+CD137+ cells (*p* = 0.012, *r* = 0.60, CI (0.15–0.84)) (Figure 2(c)).

## 4. Discussion

In this study, we focused on the possible role of costimulatory molecule CD137 in a cohort of SLE patients. Our analysis demonstrated a correlation between membrane and soluble CD137 and SLEDAI-2K values, suggesting a potential role as biomarkers for disease activity. At present, there is a lack of studies investigating the role of CD137 in SLE patients. A recent comprehensive review summarizing studies on the function of membrane and soluble CD137 in SLE highlighted the need to translate results obtained in mice to humans [6].

As known, CD137 is a costimulatory receptor expressed on activated T cells that, by binding its ligand CD137L, enhances T cells survival and proliferation and results in interleukin-2 (IL-2) secretion [8]. The role of this molecule on SLE has given contrasting results and has been investigated prevalently in murine models. Indeed, MRL/Faslpr lupus-prone mice knocked-out for CD137 showed a more aggressive disease phenotype compared to wild-type mice with significantly higher 5 months death rate [15]. Furthermore, agonist anti-CD137 antibodies improved the disease's signs and symptoms [16]. Taken together, these results seem to suggest a protective role for CD137 axis blocking in SLE murine models.

On the other hand, our results would suggest that increased expression and secretion of CD137 could trigger autoimmune response. First, we observed an overexpression of CD137 on CD3+ T cells in SLE patients compared to HDs, thus suggesting a role of CD137 in activating immune response.

Moreover, the expression of CD137 on CD4+ T cells seems to play a role as biomarker for disease activity. In fact, we found a positive correlation between the percentage of CD4+CD137+ cells and SLEDAI-2K values; accordingly, patients in remission showed significantly lower percentage of these T cells compared with patients with active disease. This correlation resulted peculiar for CD4+ cells, since not observed in CD8+ T cells. This result agrees with previous studies observing a CD4+ T-cell driven SLE pathogenesis [17]; thus, we can suppose that CD137 overexpression may contribute to the persistent activation of adaptive immune response by perpetuating costimulation signaling.

Contrarily to its membrane form, soluble CD137 plays an inhibitory role; by binding CD137L, it impedes the development of the so-called CD137-CD137L axis [18]. In this study, we observed a positive correlation between serum CD137 levels and the percentage of CD4+CD137+ cells in addition to the SLEDAI-2K values. These results also suggested for the soluble form a possible role as biomarker for disease activity.

As far as we know, only one other study evaluated serum CD137 levels in SLE patients, showing significant higher levels compared to HDs, but without providing data about potential association with disease activity [19]. Certainly, this study shows some limitations. First, the limited number of patients included in this study, not allowing the correlation with specific disease features. Furthermore, this is a cross-sectional study, requiring a future longitudinal extension to evaluate the sensitivity to change of CD137, according to treatment.

In conclusion, we could consider this analysis as a pilot and exploratory study aiming at investigating the role of both membrane and soluble CD137 in SLE. By demonstrating a correlation between this molecule and SLEDAI-2K values, we proposed CD137 as biomarkers for disease activity.

## Figures and Tables

**Table 1:** Clinical and laboratory features and ongoing treatment of the SLE cohort, expressed and percentage of patients (%).

Clinical features	
Mucocutaneous involvement	85%
Musculoskeletal involvement	80%
Serositis	20%
Kidney involvement	25%
Hematological manifestations	50%
Neurological involvement	10%
Thrombotic events	5%
Laboratory features	
Anti-dsDNA	70%
Anti-SSA/anti-SSB	50%
Anti-RNP	20%
Anti-Sm	35%
Antiphospholipid	15%
Low C3/C4 levels	60%
Ongoing therapy	
Glucocorticoids	50%
Hydroxychloroquine	80%
Mycophenolate mofetil	15%
Cyclosporin A	10%
Azathioprine	15%
Belimumab	15%
Antiplatelet therapy	25%

RNP, ribonucleoprotein.

**Table 2:** Details about all the SLE patients enrolled in the present analysis.

Patients	SLEDAI-2K	Clinical and laboratory features	Treatments
Patients with active disease			
1	6	Arthritis, low complement	GCs, MMF, BLM
2	8	Proteinuria, anti-dsDNA, low complement	HCQ, MMF
3	12	Proteinuria, hematuria, anti-dsDNA, low complement	GCs, HCQ, AZA
4	8	Mucosal ulcers, arthritis, low complement	HCQ
5	16	Rash, low complement, proteinuria, hematuria, pyuria	GCs, HCQ
6	8	Arthritis, anti-dsDNA, low complement	GCs, HCQ, MMF, BLM
7	6	Arthritis, anti-dsDNA	HCQ
8	6	Mucosal ulcers, rash, anti-dsDNA	GCs, HCQ
9	6	Rash, arthritis	HCQ, AZA
10	12	Mucosal ulcers, proteinuria, low complement, hematuria	GCs, HCQ
Patients in remission			
11	0	–	GCs, AZA
12	0	–	GCs, BLM
13	0	–	HCQ
14	0	–	HCQ
15	0	–	GCS, HCQ, CyA
16	0	–	HCQ
17	0	–	GCs, HCQ
18	0	–	HCQ, CyA
19	0	–	HCQ
20	0	–	HCQ

Note: In particular, data about ongoing manifestations concurring to SLEDAI-2K values were reported. GCs, glucocorticoids; HC, hydroxychloroquine; MMF, mycophenolate mofetil; BLM, belimumab; AZA, azathioprine; CyA, cyclosporin A.

## Data Availability

Data are available upon reasonable request.
